# Structural Study of Metakaolin-Phosphate Geopolymers Prepared with Wide Range of Al/P Molar Ratios

**DOI:** 10.3390/polym17172358

**Published:** 2025-08-30

**Authors:** Martin Keppert, Martina Urbanová, Ivana Šeděnková, Václav Pokorný, Michala Breníková, Jitka Krejsová, Vojtěch Pommer, Eva Vejmelková, Dana Koňáková, Jiří Brus

**Affiliations:** 1 Department of Materials Engineering and Chemistry, Faculty of Civil Engineering, Czech Technical University in Prague, Thákurova 7, 166 29 Paha 6, Czech Republic; brenimic@student.cvut.cz (M.B.); jitka.krejsova@fsv.cvut.cz (J.K.); vojtech.pommer@fsv.cvut.cz (V.P.); eva.vejmelkova@fsv.cvut.cz (E.V.); dana.konakova@fsv.cvut.cz (D.K.); 2 Department of Structural Analysis, Institute of Macromolecular Chemistry CAS, Heyrovského Náměstí 1888/2, 162 00 Praha 6, Czech Republic; urbanova@imc.cas.cz (M.U.); sedenkova@imc.cas.cz (I.Š.); pokorny@imc.cas.cz (V.P.)

**Keywords:** silico-alumino-phosphate geopolymers, phosphoric acid activation, Al/P molar ratio, metakaolin, solid-state NMR spectroscopy, small-angle X-ray scattering (SAXS), compressive strength

## Abstract

Geopolymers represent an innovative and environmentally sustainable alternative to traditional construction materials, offering significant potential for reducing anthropogenic CO_2_ emissions. Among these, phosphoric acid-activated metakaolin-based systems have attracted increasing attention for their chemical and thermal resilience. In this study, we present a comprehensive structural and mechanical evaluation of metakaolin-based geopolymers synthesized across a wide range of Al/P molar ratios (0.8–4.0). Six formulations were systematically prepared and analyzed using X-ray powder diffraction (XRPD), small-angle X-ray scattering (SAXS), Fourier-transform infrared spectroscopy (FTIR), solid-state nuclear magnetic resonance (ssNMR), and complementary mechanical testing. The novelty of this work lies in the integrated mapping of composition–structure–property relationships across the broad Al/P spectrum under controlled synthesis, combined with the rare application of SAXS to reveal composition-dependent nanoscale domains (~18–50 nm). We identify a stoichiometric window at Al/P ≈ 1.5, where complete acid consumption leads to a structurally homogeneous AlVI–O–P network, yielding the highest compressive strength. In contrast, acid-rich systems exhibit divergent flexural and compressive behaviors, with enhanced flexural strength linked to hydrated silica domains arising from metakaolin dealumination, quantitatively tracked by ^29^Si MAS NMR. XRPD further reveals the formation of uncommon Si–P crystalline phases (SiP_2_O_7_, Si_5_P_6_O_25_) under low-temperature curing in acid-rich compositions. Together, these findings provide new insights into the nanoscale structuring, phase evolution, and stoichiometric control of silica–alumino–phosphate geopolymers, highlighting strategies for optimizing their performance in demanding thermal and chemical environments.

## 1. Introduction

The development of sustainable construction materials is a critical component of the global effort to reduce anthropogenic CO_2_ emissions. A major contributor to these emissions is the production of Portland cement, which motivates the search for alternative binders with lower environmental impact [1]. One such alternative involves the use of chemically activated aluminosilicates, commonly referred to as geopolymers, which are formed by the reaction of an amorphous aluminosilicate precursor with an alkaline or acidic activator. While alkaline activation has been extensively studied and industrially applied, acid activation—typically utilizing phosphoric acid—has received considerably less attention, despite its potential for specialized applications [2].

Silico-alumino-phosphate (SAP) geopolymers, synthesized by acid activation of a calcined clay, have shown promising properties, including high thermal and chemical stability, making them suitable for specific applications such as thermal insulation foams [3], high-temperature resistant coatings [4], and radioactive waste immobilization matrices [5]. However, the high cost and limited availability of phosphorus compounds make these materials impractical for large-scale applications such as concrete production [6]. Optimizing SAP geopolymers for high-value, performance-driven uses requires a fundamental understanding of their structural formation mechanisms and the influence of compositional parameters (Table 1).

**Table 1:** Summary of published structural studies on SAP geopolymers; MK—metakaolin; CS—compressive strength; IC—isothermal calorimetry; TA—thermal analysis; HT—high temperature.

Ref.	Precursor	Methods	Curing	Variables	CS MPa (28 Days)	Structural Findings
[7]	Kaolinitic-illitic calcined clay	CS, XRD, NMR, FTIR, SEM	amb. 2 h; 60 °C 24 h	Al/P	6.6	Highest strength for Al/P = 1. Higher content of amorphous matter = higher strength. Tetracoordinated P, hexacoordinated Al, Si in four different environments.
[8]	Kaolinitic-illitic calcined clay	CS, XRD, NMR, FTIR, SEM	amb. 2 h; 60 °C 24 h	Al/P; particle size	34 (21 days)	SAP consists in amorphous -Si-O-P- matrix with dispersed berlinite (AlPO_4_) crystals. Finer precursor = faster polymeration, denser structure.
[9]	MK	CS, FTIR, XRD, SEM, MIP	amb. 24 h; 60 °C 24 h	Conc. of H_3_PO_4_	94	SAP consists in amorphous -Si-O-P- matrix with dispersed berlinite (AlPO_4_) crystals.
[10]	Al_2_O_3_–2SiO_2_ sol-gel powder	CS, XRD, NMR, FTIR	60 °C 3 days	Curing temp. 200–900 °C	89	Formation of -Si-O-P-O-Al- structure.
[11]	MK Al(H_2_PO_4_)_3_	CS, XRD, NMR, TA, MIP, SEM	RT, high rel. humid.	Al/P in activator	37	Formation of -Si-O-P-O-Al- structure.
[12]	MK	CS, SEM, XRPD, NMR, FTIR	60 °C 24 h	Al/P	2	Formation of Al-O-P- geopolymer dispersed in Si-O-P-O-Al- geopolymer.
[13]	MK	CS, IC, Vicat, NMR, FTIR	40 °C 24 h; 60 and 80 °C 24 h	H_3_PO_4_ conc.; P/Al	120	Major structural units of the SAP include Al-O-P, Si-O-P, Si-O-Si and unreacted MK.
[14]	MK	FTIR, XRD, HT-NMR, TA	amb. 21 d	thermal treatment	N/A	Formation of -Al-O-P- and Si-O-Al- networks and silica gel.
[15]	MK	CS, FTIR, TA, porosity, XRD, Vicat, viscosity	70 °C 72 h	water content; Al/P	78	Al/P = 1: the network is based on Al–O–P bonds with a hydrated silica network., The formation of Si–O–P and Si–O–Al bonds depends on Al/P ratio and unreacted metakaolin.
[16]	MK	CS, IC, FTIR, NMR	amb. or 60 °C	time of curing	30 (15 days)	Formation of two geopolymeric networks: -Al-O-P- and -Si-O-(Si, Al, P)
[17]	MK	XRD, FTIR, TA, NMR, IC, SEM	60 °C	time of curing	N/A	Amorphous silica is dispersed in -Si-O-Al-O-P- geopolymer.
[18]	MK	TA, XRD, NMR	20, 40, 70 °C	Al/P, HT	N/A	SAP is composed mostly of Al-O-P and Si(OH)_4_ networks.

Structural results of previous studies on SAP systems (primarily based on phosphoric acid-activated metakaolin or calcined kaolinitic clays) are summarized in Table 1. Several structural models of SAP were proposed in the literature. These include (i) a single network structure composed of Si-O-P-O-Al units [10,11]; (ii) an Si-O-P-based amorphous matrix with dispersed berlinite (trigonal polymorph of AlPO_4_) crystals [8,9]; (iii) dual network systems composed from Al-O-P and Si-O-P segments [12,14]; and (iv) a combination of Al-O-P and hydrated silica domains [15,18]. Despite these efforts, the precise structural organization of SAP geopolymers remains incompletely resolved, which we consider a research gap in the field of fundamental knowledge on SAP geopolymers.

This study aims to deepen the understanding of SAP geopolymer structures by systematically investigating the effect of Al/P molar ratio on structural evolution and mechanical performance (compressive as well as bending strength). The experimental results should contribute to the discussion of which of the above listed structural models of SAP is the closest to the real structure of this material. A set of complementary techniques—ssNMR, FTIR spectroscopy, X-ray powder diffraction (XRD), and small-angle X-ray scattering (SAXS)—was employed to characterize the local and nanoscale architecture of SAP geopolymers synthesized from a single metakaolin source and activated by phosphoric acid. Thermogravimetry was employed to characterized the nature of water molecules embedded in the SAP. The correlation between structural motifs and physicochemical properties is discussed in the context of material optimization for targeted applications. By mapping the structural transformations induced by compositional changes, this work contributes to the broader goal of advancing acid-activated binders as functional, application-specific materials.

## 2. Materials and Methods

### 2.1. Raw Materials

Commercially produced metakaolin (MK) Mefisto L05 by ČLUZ company (Nové Strašecí, Czech Republic) was used as a precursor. Its chemical composition (by XRF) and particle size parameters (obtained by laser diffraction granulometry) are given in Table 2. The density of metakaolin was 2610 kg/m^3^. The phase composition was examined by X-ray diffractometer PAnalytical Aeris (PAnalytical, Almelo, The Netherlands). Metakaolin is, as expected, highly amorphous (90%), but it also contains some impurities (quartz 4%, anatase 2%, and residuals of mica/illite crystals 4%). When the SiO_2_ content in quartz and illite and Al_2_O_3_ content in illite are taken into account, the amorphous portion of metakaolin consists of 46.2% SiO_2_ and 40.8% Al_2_O_3_. These values were used when the SAP mixtures of various Al/P molar ratios were prepared. The corresponding molar ratio of Si and Al is 0.49:0.51, which is close to dehydroxylated kaolinite (molar ratio ideally 1:1). Phosphoric acid (p.a.) was in the form of an 85% solution.

### 2.2. Prepared SAP

A set of SAP geopolymers was prepared from MK and an 85% H_3_PO_4_ solution (Table 3). The labels refer to Al/P molar ratio in activated matter (when one assumes that 40.8% by mass of MK is formed by amorphous Al_2_O_3_). The amount of water was adjusted to a consistency of a 160 mm spill (according to EN 1015 standard). An aqueous solution of the acid was mixed with the MK. The liquid mixture was poured into 20 × 20 × 160 mm^3^ molds made from Hakorit (UHMWPE). The samples were kept for 24 h at lab temperature (20 ± 2 °C) and then cured at 60 °C for another 24 h. The molds with samples were covered by plastic foil in order to eliminate possible quick drying and cracking. Then the samples were demolded and kept in laboratory conditions. The compressive and flexural strengths were determined 7 days after the casting, and then other analyses were performed. The stability of the sample after one week of curing was confirmed by an independent experiment.

### 2.3. Methods

The elementary composition of metakaolin was acquired by an XRF spectrometer ARL 9400 XP (Thermo Scientific, Ecublens, Switzeland). The phase composition of metakaolin and SAP was evaluated by X-ray diffraction with the help of a PAnalytical Aeris diffractometer (PAnalytical, Almelo, The Netherlands). equipped with a Co_Kα_ tube operating at 7.5 mA and 40 kV. Data were evaluated by Rietveld refinement performed by Profex software (ver. 5.0.2) [19]. The content of amorphous matter was determined by the employment of an internal standard (20% of ZnO). The compressive and three-point bending strengths were measured by a loading device EU40 (Heckert, Zella-Mehlis, Germany). Three prisms were prepared per each mixture; thus, three values of bending and six values of compressive strength were acquired. The standard deviation of the mechanical tests was depicted by means of error bars. Bulk density of SAP geopolymers was obtained with the help of mass and dimensions of prismatic specimens. Specific gravity of materials was determined by a helium pycnometer Pycnomatic ATC (Thermo Electron, Milan, Italy). Porosity was calculated with the help of bulk density and specific gravity. The simultaneous TG/DSC was performed by a Netzsch STA 449 F3 Jupiter (Netzsch, Selb, Germany). Argon atmosphere and a 10 °C/min heating rate were used. Evolved gas analysis was performed by a connected mass spectrometer (Netzsch, Selb, Germany). Pore size distribution was measured by mercury intrusion porosimetry (MIP) (devices Pascal 140 and 440 made by Thermo Electron, Milan, Italy). The SEM-EDS (energy-dispersive X-ray spectroscopy) analysis was performed by a PhenomXL microscope (Phenom-World, Eindhoven, The Netherlands) in BSE mode with an acceleration voltage of 15 kV.

FTIR spectra of the powdered samples were measured in the KBr pellets. The spectra were recorded on a Nicolet Nexus 6700 spectrometer (Thermo Fischer Scientific, Madison, WI, USA) equipped with a DTGS detector. A total of 64 scans at a 2 cm^−1^ resolution were accumulated for each spectrum. Small-angle X-ray scattering (SAXS) experiments were performed using a pinhole camera (modified Molecular Metrology system; Rigaku, Tokyo, Japan) attached to a microfocused X-ray beam generator (MicroMax 003, Rigaku, Tokyo, Japan) operated at 50 kV and 0.6mA (30 W). The camera was equipped with a vacuum-compatible version of a Pilatus3 R 300 K hybrid photon-counting detector (Dectris, Baden, Switzerland). A setup upgrade made by SAXSLAB (Søborg, Denmark) enabled the detector to stay aligned with the beam. Sample-to-detector distance was set to 1000 mm, and an exposure time of 3 h was used for the experiments.

Solid-state NMR spectra were measured at 11.7 T using a Bruker Avance 500 WB/US NMR spectrometer (2013; Bruker Corporation, Billerica, MA, USA) in a double-resonance 4 mm probe-head. All ^27^Al, ^29^Si, and ^31^P MAS NMR spectra were acquired using direct polarization experiments (single pulse excitation). The ^27^Al MAS NMR spectra were acquired at 130.33 MHz, spinning frequency was *ω*_r_/2π = 11 kHz, 20° pulse width was 1 µs, recycle delay was 2 s, and the number of scans was 256. The ^27^Al chemical shift scale was calibrated with an external standard AlNO_3_ at 0.0 ppm. The ^29^Si MAS NMR spectra were acquired at 99.37 MHz using a conventional 90° pulse and spinning frequency of *ω*_r_/2π = 7 kHz. The recycle delay was optimized and tested using the values 2, 7, and 30 s. It was found out that the recycle delay of 2 s is sufficiently long to obtain undistorted ^29^Si MAS NMR spectra. The number of scans were about 20 k. The ^29^Si chemical shift scale was calibrated with an external standard M_8_Q_8_ (the signal with the lowest frequency was set at −109.8 ppm). The ^31^P MAS NMR spectra were acquired at 202.404 MHz, a conventional 90° pulse, spinning frequency of *ω*_r_/2π = 10 kHz, recycle delay of 10 s, and 128 scans. The ^31^P scale was calibrated with an external standard CaHPO_4_ set at −0.6 ppm.

## 3. Results

To study the effect of Al/P molar ratio on the formation and structure of metakaolin-based phosphate geopolymers, a series of six formulations were synthesized (Table 3). The systematic characterization and evaluation using a wide range of experimental techniques aimed to correlate structural evolution with macroscopic material properties.

### 3.1. Physical Properties

#### 3.1.1. Mechanical Properties and Basic Physical Properties

**Figure 1:**
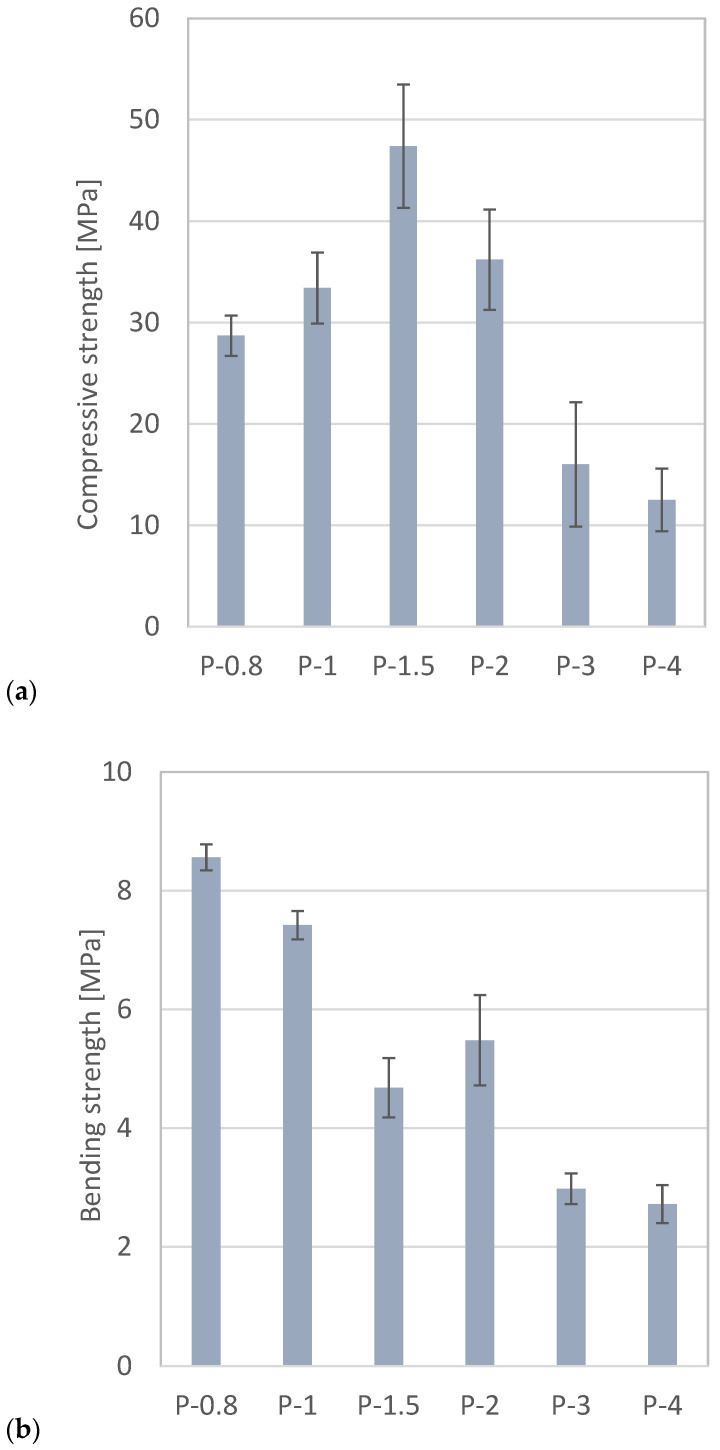
Compressive (**a**) and flexural (**b**) strength of SAP geopolymers measured in 7 days.

Compressive and flexural strength are key indicators of material performance. The values measured after 7 days of curing are presented in Figure 1. The published compressive strength of the synthesized SAP geopolymers is in the broad range of 2–120 MPa (Table 1); the obtained results fall within this broad range. The highest compressive strength was achieved in the sample with an Al/P molar ratio of 1.5, while previous studies have often reported an optimum at Al/P = 1. Some values of compressive strength have high standard deviations. It implies inhomogeneity of samples, likely caused by imperfect mixing and casting of fresh SAP to molds. The flexural strength of the SAP geopolymers decreased with increasing Al/P ratio, meaning that samples containing a higher amount of phosphoric acid exhibited better flexural performance (Figure 1b). This inconsistency of compressive and flexural performance, not usual in inorganic materials, was caused by excessive phosphoric acid—sample P-0.8 featured apparent plastic or ductile behavior.

**Figure 2:**
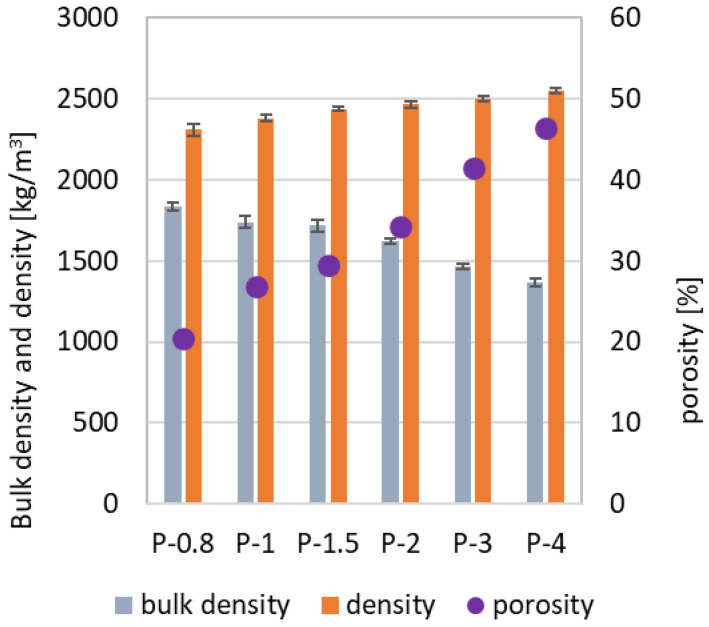
Porosity, bulk density, and specific gravity of SAP geopolymers.

**Figure 3:**
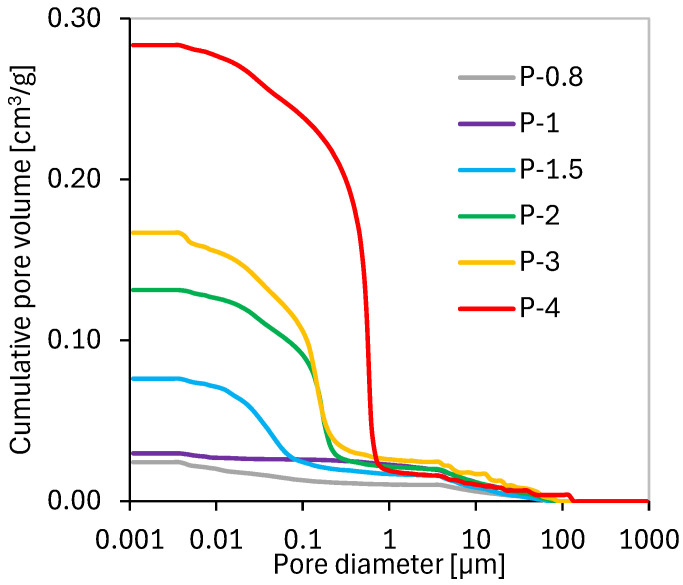
Cumulative porosimetry curves of SAP geopolymers.

The mechanical performance of porous inorganic materials, including geopolymers, is closely related to their pore structure; higher porosity means lower strength. To assess this relationship, the bulk density, density, and porosity of the SAP samples were measured (Figure 2). The density of the sample with the highest Al/P ratio (P-4), i.e., with the lowest phosphoric acid content, was close to that of the original metakaolin (2552 kg·m^−3^ vs. 2610 kg·m^−3^). As the Al/P ratio increased, the density increased, and the bulk density decreased, reflecting significant changes in the chemical structure. The resulting value of porosity ranged from approximately 20% in P-0.8 to 46% in P-4. The pore size distribution, determined by MIP (Figure 3), was unimodal in all cases. The threshold pore diameter increases with Al/P ratio from ~0.1 µm in the most acid-rich systems to ~1 µm in the acid-deficient specimens. The most compact samples (P-0.8 and P-1) exhibited no distinct threshold pore diameter, but it has to be kept in mind that MIP does not detect pores smaller than 4 nm or larger than 100 µm.

Sample P-1.5 exhibited the highest compressive strength despite having an intermediate porosity within the tested series. This implies that the mechanical performance of SAP geopolymers is influenced not only by their physical microstructure but also by the chemical nature and connectivity of the geopolymer network formed during acid activation. Another observed trend is that the total pore volume (i.e., porosity and bulk density) is clearly linked to the amount of water added to the mixture (in order to achieved proper consistency—Table 3).

#### 3.1.2. Scanning Electron Microscopy

**Figure 4:**
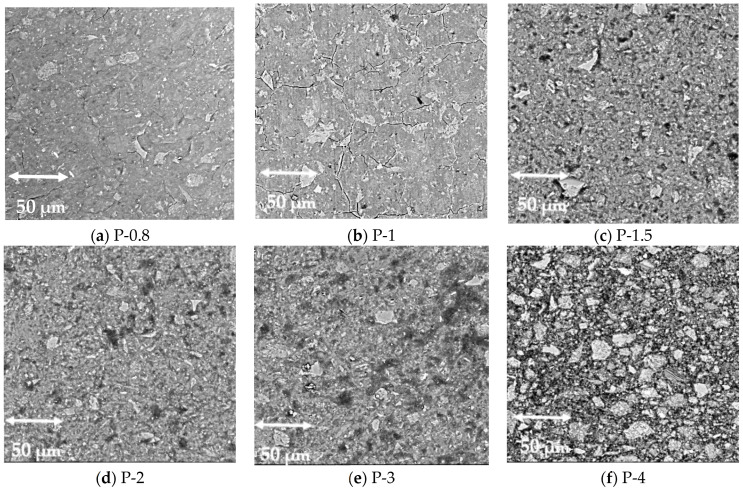
SEM images of SAP geopolymers. The increasing porosity of samples with increasing Al/P molar ratio can be observed. (**a**) P-0.8; (**b**) P-1; (**c**) P-1.5; (**d**) P-2; (**e**) P-3; (**f**) P-4.

SEM imaging was used to investigate the microstructural evolution of SAP geopolymers with varying Al/P ratios (Figure 4). A clear trend was observed: samples with low Al/P ratios (P-0.8, P-1) exhibited dense, compact structures with limited porosity, while those with higher Al/P ratios (P-3, P-4) exhibited increasingly loose and porous networks. These observations are consistent with the MIP data and further confirm that porosity increases with Al/P ratio.

**Figure 5:**
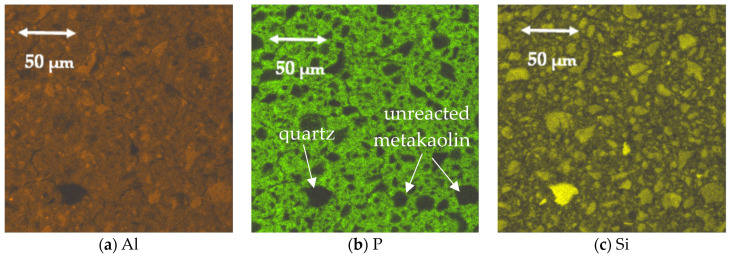
Results of EDS analysis of P-1.5 sample. (**a**) Al; (**b**) P; (**c**) Si.

To estimate and visualize the present phases, energy-dispersive X-ray spectroscopy (EDS) mapping was performed on sample P-1.5 (Figure 5). Al, P, and Si distribution maps enabled us to identify quartz grains (Si present, Al and P absent) and unreacted metakaolin particles (Al and Si present, P absent). The continuous matrix, showing signals for Al, Si, and P, confirmed the formation of a geopolymeric phase containing all three elements.

#### 3.1.3. Thermogravimetry and Differential Scanning Calorimetry

**Figure 6:**
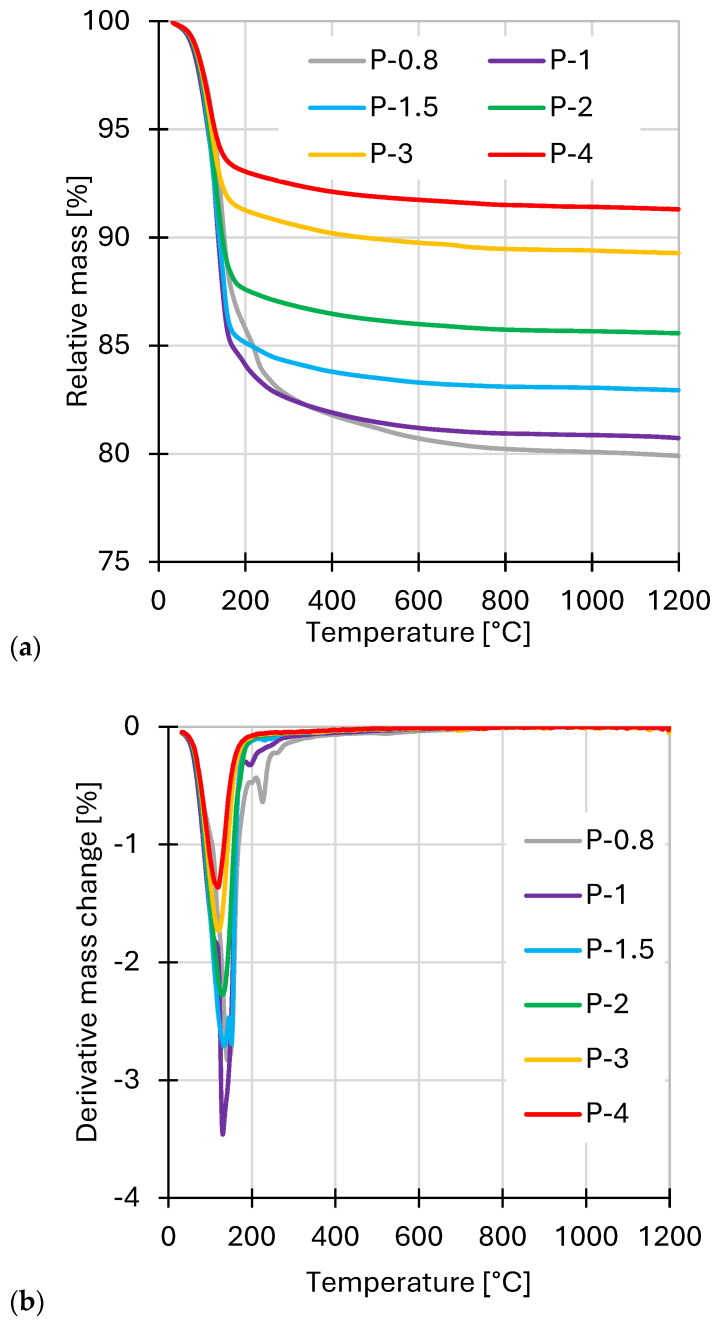

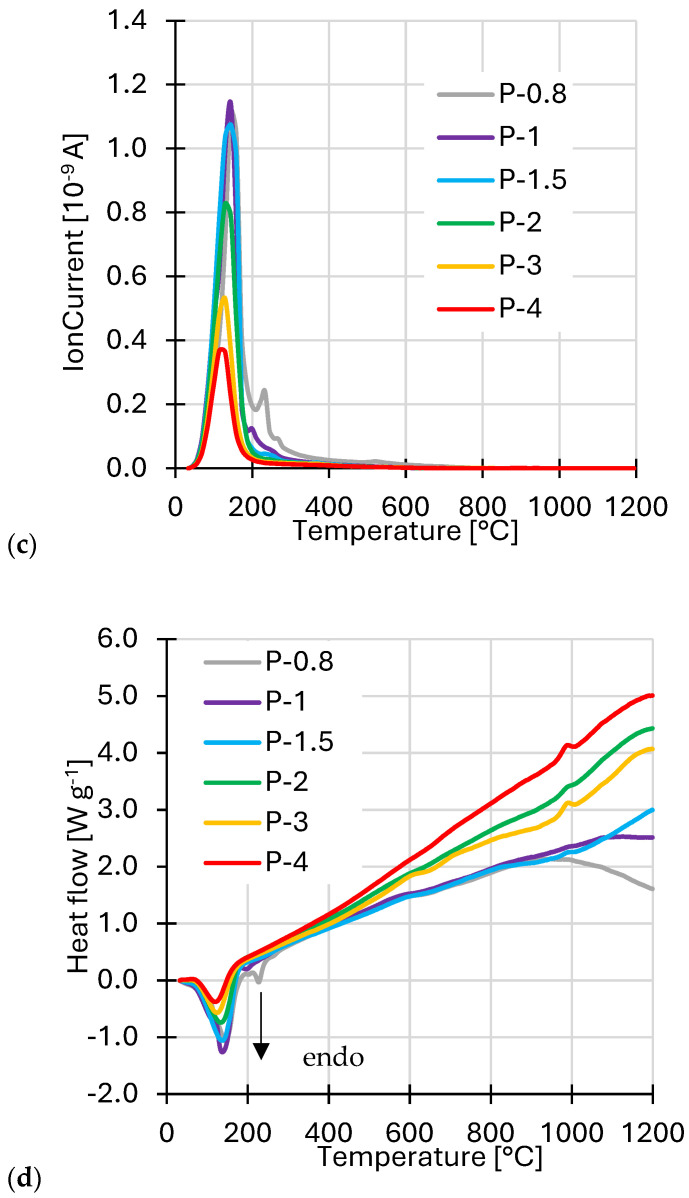
Results of SAP: (**a**) thermogravimetry (TG); (**b**) its derivative curves (dTG); (**c**) evolved gas analysis for *m*/*z* = 18 (water); and (**d**) differential scanning calorimetry (DSC).

Simultaneous thermogravimetry (TG) and differential scanning calorimetry (DSC) was used to evaluate the thermal stability and bound water content of the SAP geopolymers (Figure 6). The SAP samples were subjected to continuous heating up to 1200 °C under an argon atmosphere without prior drying. The observed mass loss (in all formulations) was entirely due to the evolution of water, based on the evolved gas analysis where just *m*/*z* = 18 was detected (Figure 6c).

The majority of the water release occurred between 80 and 130 °C, resulting in the dehydration of physical bound water and weakly associated structural water molecules. Notably, the samples with the lowest Al/P ratios (P-0.8 and P-1) exhibited an additional, distinct mass loss at higher temperatures (175 °C and 225 °C, respectively). As the Al/P molar ratio increased, the total mass loss due to water evaporation decreased, indicating a lower content of hydrated species in acid-deficient formulations. The absence of significant mass loss above ~400 °C in all samples confirms that dehydroxylation processes (i.e., the removal of structural −OH groups) were absent in SAP. The dehydration was obviously apparent also in DSC curves by endothermic heat flow (Figure 6d). Therefore, the observed thermal behavior may be supposed to be dominated just by dehydration, not by dehydroxylation. An exothermic process was indicated at 980 °C in metakaolin-rich mixtures as well. It implies crystallization of a new phase, likely a spinel from surplus metakaolin, but high-temperature transformations were beyond the scope of this study.

### 3.2. Structure of SAP Geopolymers

Understanding the physical performance of SAP geopolymers requires a detailed insight into their chemical architecture. Given the predominantly amorphous nature of the studied SAP materials, a multi-technique approach has been used to resolve their structural characteristics. While X-ray diffraction (XRD) provides valuable information about crystalline phases, its ability to characterize the amorphous matrix is limited. This limitation is complemented by small-angle X-ray scattering (SAXS), which reveals nanoscale heterogeneities in the material, although it does not provide insight into chemical bonding. Fourier-transform infrared (FTIR) spectroscopy provides data on vibrational modes of functional groups, while solid-state nuclear magnetic resonance (ssNMR) delivers the most detailed information on the local atomic environments and coordination of Al, Si, and P atoms. The following section presents the structural findings obtained through this multi-technique approach.

#### 3.2.1. XRD and SAXS

**Figure 7:**
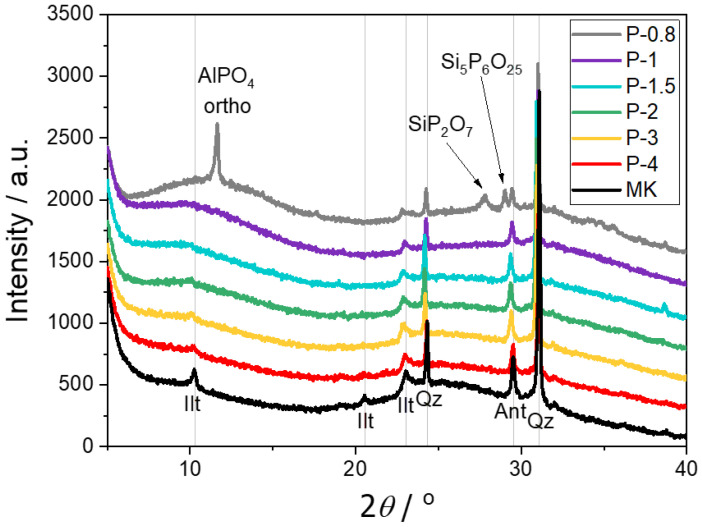
X-ray powder diffractograms of metakaolin (MK) and SAP geopolymers. Positions of peaks present in metakaolin are highlighted by vertical lines. Ilt—illite; Qz—quartz; Ant—anatase.

**X-ray diffraction (XRD)** confirmed the predominantly amorphous character of all SAP samples, with crystalline reflections corresponding in most samples only to residual phases from the metakaolin precursor—quartz, anatase, and illite (Figure 7). The only exception was sample P-0.8, which contained a high amount of phosphoric acid and exhibited several newly formed crystalline phases. The most intensive diffraction peak at 11.6° 2θ corresponded to orthorhombic modification of AlPO_4_ (ICCD 04-015-7184). While the trigonal polymorph (berlinite) is commonly reported in SAP systems [10,11], its detection is challenging due to its isostructural similarity to quartz, which is a common impurity in metakaolin and other clay-based precursors. Nevertheless, Rietveld refinement of the P-0.8 diffractogram revealed the presence of approximately 2% of berlinite (ICCD 04-009-5761) in addition to quartz (COD 9009666). Two additional crystalline phases were also identified in P-0.8: silicon pyrophosphate (SiP_2_O_7_, ICCD 04-015-4766) with a peak at 27.8° 2θ and a mixed silicon-phosphorus oxide (Si_5_P_6_O_25_, ICCD 04-011-0824) at 29° 2θ. This unusual compound (corresponding to 5SiO_2_·3P_2_O_5_) has previously been synthesized from silica gel and phosphoric acid and annealed at 400 °C [20].

Despite the presence of these crystalline phases, P-0.8 remained largely amorphous, as indicated by the characteristic broad humps in its diffractogram (Figure 7). The content of crystalline phases in SAP was determined using an internal standard (ZnO) (Table 4). It has to be noted that these values should be understood first of all as indication that the materials are highly amorphous (the accuracy of such determination based just on XRD is low). The sample P-0.8 was solely the one which contained new crystalline phases and reached the crystallinity (21%), while the rest of the SAPs contained just an amorphous phase with impurities from metakaolin.

**Figure 8:**
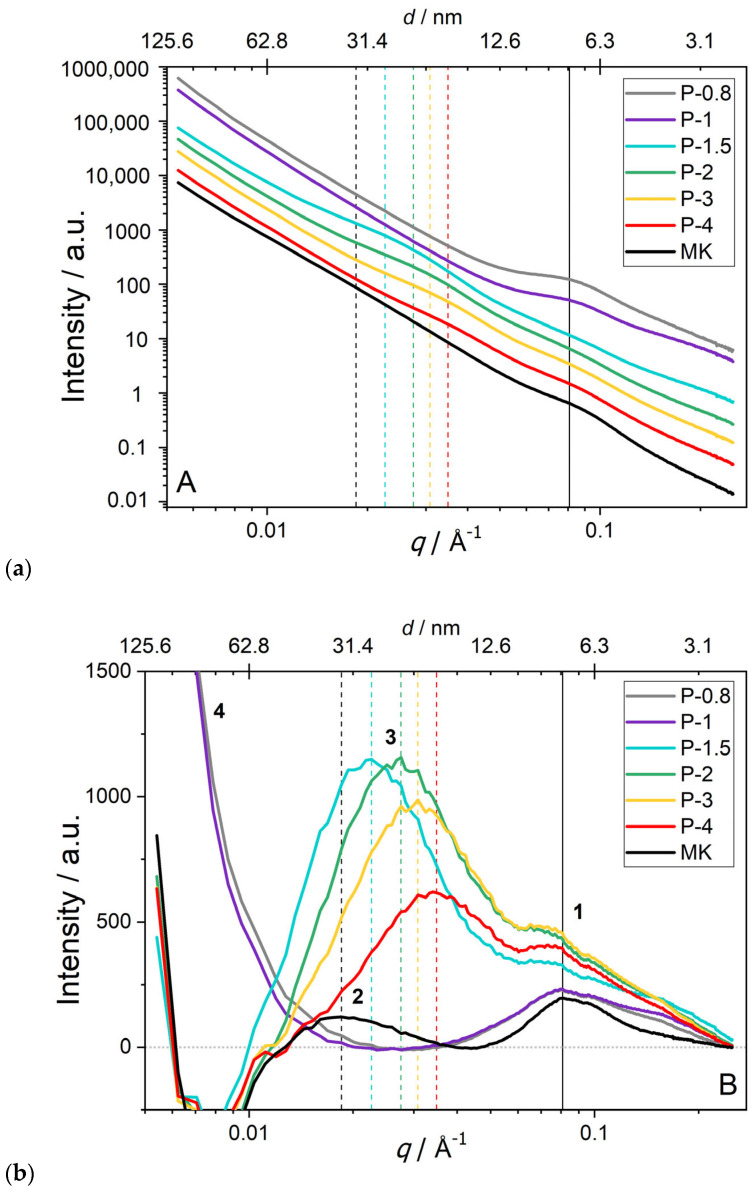
SAXS data recorded on metakaolin and SAP geopolymers. Positions of peaks are highlighted by vertical lines; (**a**) standard log-log plot; (**b**) semi-log plot after subtracting a power-law function corresponding to the general slope of the SAXS curves.

**Small angle X-ray scattering** (SAXS, Figure 8) was used as a complementary technique to mercury intrusion porosimetry (MIP, Figure 3). The scattering vector, q, is defined as *q* = 4π/λ·sin(θ), where λ = 1.54 Å is the radiation wavelength and 2θ is the scattering angle. Consequently, SAXS probes electron-density fluctuations and thus reports characteristic nanoscale correlation lengths associated with pores, gel clusters, or phase boundaries. Accordingly, from the position of a broad maximum at *q_peak_*, we estimate a characteristic “feature size” *d* ≈ 2π/*q_peak_*. We deliberately use “feature size” rather than “pore size” unless independent evidence identifies the feature as a pore. In metakaolin, two peaks at *q* ≈ 0.09 and 0.018 Å^−1^ correspond to features of ~8 nm and ~33 nm, respectively (denoted as peaks 1 and 2 in Figure 8b), and the features have also been observed in earlier SAXS studies of metakaolin [21]. These features persisted after acid activation. During geopolymerization, new SAXS maxima emerged in samples P-1.5 to P-4, corresponding to nanoscale domains or clusters whose diameters decreased from ~28 nm (P-1.5) to ~18 nm (P-4). In contrast, acid-rich samples P-0.8 and P-1 displayed a pronounced low-q upturn, indicating the presence of larger-scale motifs outside the measurement window (d > 50 nm), consistent with mean pore sizes obtained by MIP (Figure 3). We note that MIP and SAXS probe complementary aspects of porosity: MIP quantifies accessible, connected pores (~4 nm to 100 µm), whereas SAXS senses all electron-density heterogeneities, including closed porosity and nanoscale gel domains. Consequently, qualitative trends can agree (e.g., larger-scale heterogeneity at low *q* vs. MIP-inferred coarsening), but a strict one-to-one pore size correspondence is not expected.

#### 3.2.2. FTIR Spectroscopy

**Figure 9:**
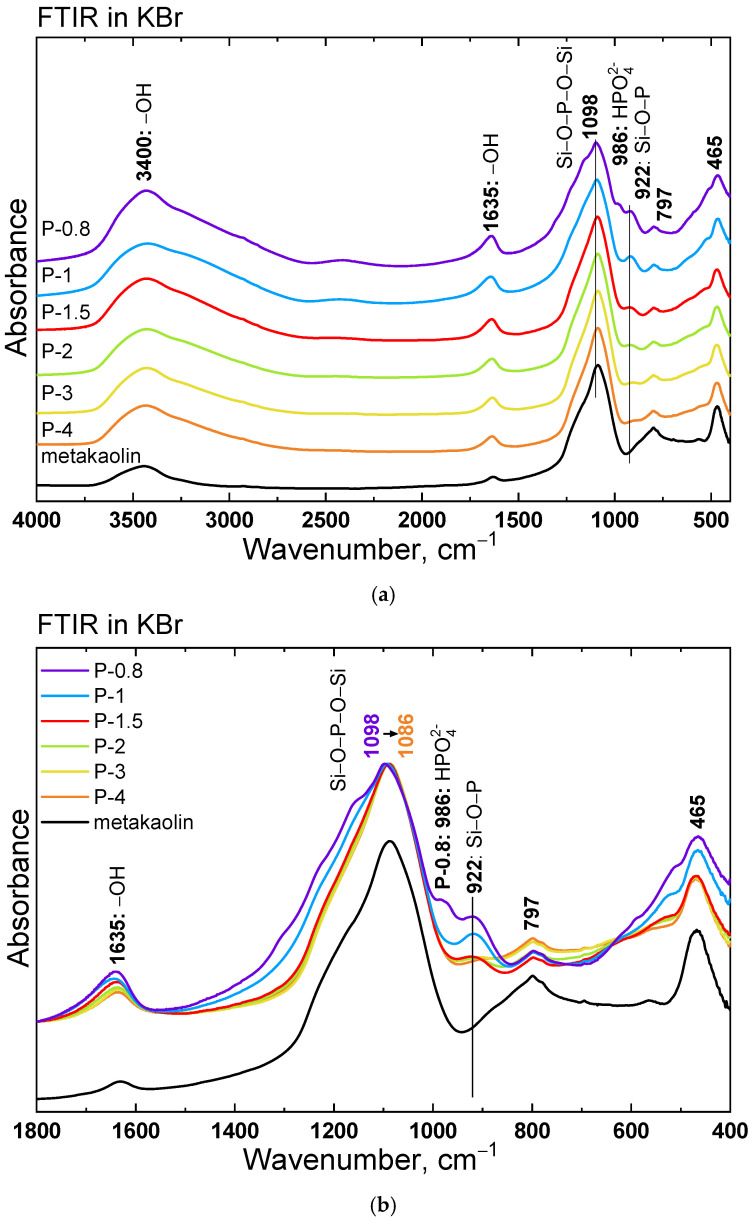
FTIR absorption spectra of metakaolin and SAP geopolymers prepared with various H_3_PO_4_ contents, measured in KBr tablets; (**a**) full spectral range; (**b**) detail of 1900–400 cm^−1^.

FTIR spectra, collected to monitor structural changes in metakaolin and SAP geopolymers across different Al/P molar ratios, are summarized in Figure 9. The FTIR spectrum of metakaolin displayed typical features of aluminosilicate materials. The main band at 1088 cm^−1^ corresponds to asymmetric stretching vibrations of Si–O–T units (T = Si or Al) [22]. Additional bands at 800 cm^−1^ and 465 cm^−1^ are attributed to symmetric Si–O vibrations in amorphous silica/quartz and Si–O–T bending modes, respectively. Broad absorption near 3400 cm^−1^ and a weaker band at 1635 cm^−1^ reflect –OH stretching and H–O–H bending vibrations from adsorbed water, respectively, consistent with the presence of illite/mica impurities identified by XRD.

Upon acid activation, several changes were observed in the FTIR spectra of SAP samples. The –OH band around 3400 cm^−1^ increases significantly after activation, indicating incorporation of water into the geopolymer gel structure. As the band is broad, we can expect the involvement of various types of H-bonds in the water envelope. The intensity of both -OH-assigned bands decreased with increasing Al/P ratio, consistent with TGA results showing lower water content in acid-deficient systems.

Spectra of samples with low Al/P (P-0.8 and P-1) show a new band at about 2420 cm^–1^ with distinct shoulders at 1160 and 1125 cm^−1^ and a band at 986 cm^−1^, attributed to excess phosphoric acid and the presence of HPO_4_^2−^ anions. A new band at ~922 cm^−1^ appears in all SAP samples, indicating the formation of Si–O–P linkages. The features associated with unreacted H_3_PO_4_ disappear with higher Al/P ratio, suggesting that all phosphoric anions are involved in Si–O–P linkages in sample P-1.5. The intensity of the 800 cm^−1^ band increases with Al/P ratio, reflecting the presence of unreacted or partially reacted silica due to limited acid availability. Additionally, enhanced absorption near 465 cm^−1^ in P-0.8 and P-1 supported structural reorganization of the Si–O–T network. The main Si-O-T band shifts from 1098 cm^–1^ in the spectrum of the P-0.8 sample to 1086 cm^−1^ of the P-4 sample. This red shift suggests the formation of Si-O-P-O-Si linkages between Si-O of the dealuminated metakaolin layer and P-O groups from phosphoric acid, particularly in the samples with a higher content of phosphoric acid.

#### 3.2.3. Solid-State NMR Spectroscopy

Solid-state MAS NMR spectroscopy provided detailed insights into the coordination environment of Al, Si, and P atoms in both raw and activated materials [17,23,24]. In general, the signal assignment in the ^27^Al and ^29^Si MAS NMR spectra of aluminosilicates is very complicated due to the large number of strongly overlapping NMR signals [23]. Especially in the ^29^Si MAS NMR spectra, a great problem follows from the fact that an increasing degree of condensation of silanol groups leads to the gradual decrease in the ^29^Si NMR chemical shift, whereas the same trend is observed with a decreasing degree of aluminum substitution in the second coordination sphere of the siloxane units. That is why in many cases, the resulting solid-state NMR spectra are of the low resolution, and the reported signal assignment must be considered just as tentative.

**Figure 10:**
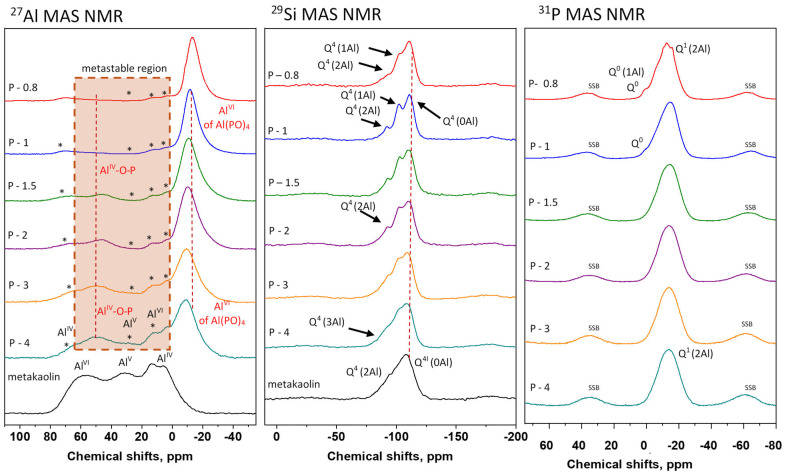
^27^Al, ^29^Si, and ^31^P MAS NMR spectra of raw metakaolin in the synthesized SAP geopolymers. Asterisks in ^27^Al MAS NMR spectra correspond to signals obtained in the initial metakaolin.

Typically, the aluminum sites of metakaolin [25,26] exhibit a very broad distribution involving interconnected units in tetrahedral (ca. 34%), pentagonal (ca. 28%), and hexagonal (ca. 38%) coordination (Al^IV^, Al^V^, and Al^VI^ units) resonating at ca 57, 31, and 13 and 5 ppm, respectively (Figure 10, ^27^Al MAS NMR spectrum). As follows from the corresponding ^29^Si MAS NMR spectrum, the extent of chemical coupling of SiO_4_^4−^ tetrahedra with aluminum species in metakaolin is relatively low because the predominantly uncombined/unsubstituted silica tetrahedra Q^4^(0Al) and partially combined silica units Q^4^(1Al), Q^4^(2Al), and Q^4^(3Al) resonating at ca −111, −103, and −94, and −86 ppm, are present. Quantitatively these units represent roughly 43, 33, 17, and 6%, respectively. Part of the Q^4^(0Al) are found in the crystalline quartz.

After activation with phosphoric acid, significant structural changes occurred (Figure 10). The ^27^Al MAS NMR spectra of SAP samples revealed two new signals at ~50 ppm and −13 ppm, attributed to Al^IV^–O–P and Al^VI^–O–P species (marked by vertical dashed lines in Figure 10). Formation of these new alumino-phosphate entities is accompanied by a considerable consumption of original Al^IV^, Al^V^, and Al^VI^ species of metakaolin (the corresponding ^27^Al MAS NMR signals are marked by asterisks), when at the highest P/Al ratio (P-0.8), only traces of the original Al^IV^ and Al^VI^ units are detected, while the metastable Al^V^ species are completely consumed. Interestingly, the amount Al^IV^-O-P structural fragments is higher at higher Al/P ratios (20% of Al units in the P-4 system and nearly zero in the P-0.8 system). In contrast, the signal at −13 ppm (aluminum sites in octahedral coordination in Al^VI^-O-P) segments systematically increases with the decrease in Al/P ratio from ca. 40% in the P-4 system up to ca. 80% in the geopolymer P-0.8. This clearly shows that the majority of original metakaolin units Al^IV^, Al^V^, and Al^VI^ together with Al^IV^-O-P units are transformed almost exclusively to the Al^VI^-O-P structures when a sufficient amount of phosphorus is available. Simultaneously with the conversion of the majority of Al structural units toward the Al^VI^-O-P units, a clear narrowing of the main ^27^Al signal at ca. −13 ppm was observed. This indicates a remarkable increase in structural uniformity of the corresponding Al^VI^-O-P fragments. In this respect, it can be supposed that there is a possible formation of some crystalline or protocrystalline domains in systems with high phosphorus content (i.e., low Al/P molar ratio). This finding aligns well with the XRD results of the P-0.8 mixture.

The extensive redistribution of building blocks in the aluminosilicate matrix toward the more uniform crystalline-like arrangement is confirmed by the narrowing of ss-NMR signals in the ^29^Si MAS NMR spectra recorded for the systems with a high phosphorus content (Figure 10). For instance, look at the ^29^Si MAS NMR spectrum recorded for the P-1.5 and P-1 systems. In this context, it is worth noting that the observed signal narrowing reaches its maximum at Al/P = 1, which implies that this system reaches the most ordered arrangement among all mixes. Inspection of the recorded ^29^Si MAS NMR spectra also revealed that the quantitative composition of the aluminosilicate matrix of the synthesized geopolymers evolved continuously with the increase in the amount of phosphoric acid in the reaction mixture. This is directly displayed by the systematic reduction (from MK to a high P-containing system, P-0.8) of the intensity of signals reflecting highly substituted silica segments Q^4^(2Al) and Q^4^(3Al). Specifically, in the P-4 system, the Q^4^(3Al):Q^4^(2Al):Q^4^(1Al):Q^4^(0Al) ratio is 3:15:32:50, whereas in the P-1 system, this ratio markedly shifts to 1:7:36:56. This observation suggests that, at higher Al/P ratios, a considerable portion of the silica framework—approximately 15–20%—remains in the form of highly substituted Q^4^(3Al) and Q^4^(2Al) units, indicative of unreacted metakaolin residues. Conversely, lower Al/P ratios result in partial dealumination of the aluminosilicate matrix. The content of highly substituted silica building blocks Q^4^(3Al) and Q^4^(2Al) is reduced to about one-half of the original values.

The ^31^P MAS NMR spectra of SAP geopolymers contained just Q^1^(2Al) sites. A trace of Q^0^ signal is visible in P-0.8 and P-1, which indicates the excess of phosphoric acid in the mixture.

## 4. Discussion

The highest compressive strength was obtained for an Al/P molar ratio of 1.5. FTIR and ssNMR analyses showed that all the added phosphoric acid was consumed in this sample. The samples with lower Al/P ratios (P-0.8, P-1) contained residual unreacted H_3_PO_4_ (FTIR, ssNMR), while those with higher Al/P ratios (P-2 to P-4) showed evidence of unreacted metakaolin (SEM).

The structural results supporting and explaining this finding may be summarized as follows:

- The higher relative amount of phosphoric acid (i.e., lower Al/P) means: lower porosity, lower specific gravity, high flexural strength, and high amount of water molecules incorporated in the activation product.
- The high Al/P is inevitably linked with higher water dose, which also contributed to the higher porosity.
- Acid activation induced partial dealumination of the aluminosilicate framework of metakaolin. The resulting geopolymer matrix was dominated by unsubstituted Q^4^(0Al) silicon sites, followed by Q^4^(1Al) species.
- Al^VI^-O-P units were the dominant aluminum species in SAP systems with a good mechanical strength.
- Phosphorus was mainly incorporated as Q^1^(2Al) units, with minor traces of Q^0^ observed only in acid-excess samples (P-0.8 and P-1).
- All samples were predominantly amorphous, with the exception of P-0.8, which showed partial crystallization of AlPO_4_ polymorphs, SiP_2_O_7_, and Si_5_P_6_O_25_.
- SAXS revealed the emergence of new structural domains around 20–30 nm, likely reflecting phosphate-induced clustering or phase separation.
- Crystallization of new phases occurred only in highly acid-rich samples (i.e., low Al/P).
- The SAP matrix contained Al, Si, and P atoms more or less evenly distributed.

Previous studies have typically reported the optimal Al/P molar ratio for SAP strength to be around 1.0–1.2 [10,15,16,18], although some variation exists depending on precursor composition and processing parameters. In this study, an Al/P ratio of 1.5 yielded the highest compressive strength after 7 days, which correlates with the complete consumption of phosphoric acid and the formation of a well-connected polymeric network, as shown by FTIR and ssNMR. Interestingly, the excess of phosphoric acid in samples P-0.8 and P-1, although detrimental to compressive strength, led to an improvement in flexural strength. This effect has not been reported in the literature. Although the porosity of samples P-0.8 and P-1 was significantly lower than that of P-1.5, their compressive strength was still inferior. This clearly indicates that porosity alone does not control the mechanical performance of SAP systems; instead, the chemical nature and connectivity of the geopolymer framework play a dominant role.

Previously published ideas on SAP structure have been summarized in the Introduction (Table 1). In relation to existing structural models, our experimental results can be interpreted as follows:

- **Single-network Si–O–P–O–Al with dispersed amorphous silica:** The ^29^Si MAS NMR spectra showed a trend towards Q^4^(0Al) sites, consistent with amorphous silica formation. Minor amounts of Si–O–P linkages were identified by FTIR in acid-rich samples, and crystalline SiP_2_O_7_ and Si_5_P_6_O_25_ were detected in P-0.8 by XRD. However, no ^31^P MAS NMR signal around −33 ppm, characteristic of SiP_2_O_7_, was observed [27]. Thus, this structural motif is present only in limited quantities.
- **Si–O–P geopolymer with dispersed berlinite (AlPO_4_):** Berlinite was detected in very small amounts just in sample P-0.8. However, given its structural similarity to quartz and the overwhelming amorphous character of the samples, this cannot be considered the dominant structural component.
- **Dual-network model (Al–O–P and Si–O–P / Si–O–P–O–Al):** Strong evidence from ^27^Al MAS NMR supports the presence of Al^VI^–O–P domains. A coexisting, albeit minor, contribution of Si–O–P domains is also plausible.
- **Al–O–P network with hydrated silica:** This structural model aligns well with our findings. Dehydration processes observed by TGA (80–130 °C) correspond to water release from amorphous hydrated silica. The supplementary experiment showed that heating P-1.5 to 200 °C led to crystallization of tridymite and cristobalite, confirming the presence of amorphous hydrated silica gel in the SAP [28].

In summary, the SAP systems studied here consist of a geopolymeric network based predominantly on Al^VI^–O–P linkages and hydrated silica domains, the latter formed through metakaolin dealumination. Minor Si–O–P linkages are likely to be present, especially in phosphorus-rich systems.

## 5. Conclusions

The present study delivers a comprehensive and integrative structural analysis of metakaolin–phosphoric acid-based silico-alumino-phosphate (SAP) geopolymers synthesized across a broad Al/P molar ratio spectrum (0.8–4.0). Beyond reaffirming known reaction motifs, our work contributes several novel insights: the systematic mapping of composition–structure–property relationships under controlled conditions; the rare application of SAXS to reveal composition-dependent nanoscale domains; the discovery of a stoichiometric window at Al/P ≈ 1.5, where complete acid consumption yields a homogeneous Al^VI^–O–P network and maximum compressive strength; the previously overlooked divergence between compressive and flexural performance in acid-rich systems; and the detection of uncommon Si–P crystalline phases forming under low-temperature curing. Together, these findings establish an integrated structural model that links phosphorus content and stoichiometry to nanoscale organization and macroscopic behavior.

Across the studied compositions, the Al/P ratio of 1.5 emerged as a critical balance point, enabling complete reaction and the development of a cohesive network that provided superior compressive strength. In contrast, acid-rich systems exhibited a distinctive mechanical response in which compressive strength declined but flexural strength improved, reflecting the role of hydrated silica domains generated by metakaolin dealumination. At higher Al/P ratios, increasing porosity further diminished flexural performance, while the overall amorphous nature of the binders was occasionally complemented by the crystallization of AlPO_4_ polymorphs and rare Si–P phases. These observations confirm that SAP structures are governed by the interplay of Al^VI^–O–P frameworks and hydrated silica domains, whose relative prominence determines mechanical integrity.

By clarifying these nanoscale-to-macroscale relationships, this study not only deepens the fundamental understanding of SAP geopolymer chemistry but also provides practical strategies for tailoring their properties through stoichiometric control. The demonstrated capacity to optimize reaction completeness, nanoscale domains, and phase evolution underscores the potential of SAP geopolymers as sustainable, high-performance binders. Their tunable structure makes them highly suitable for demanding applications such as fire-resistant coatings, chemically robust mortars, and waste immobilization matrices, reinforcing their position as advanced alternatives in environmentally responsible construction.

## Figures and Tables

**Table 2:** Chemical composition (% by weight) and granulometry of metakaolin.

SiO_2_	Al_2_O_3_	Fe_2_O_3_	CaO	MgO	K_2_O	TiO_2_	LOI	d_50_ (µm)	d_90_ (µm)
51.8	41.9	1.1	0.1	0.9	0.9	1.7	1.0	2.8	10.1

**Table 3:** Composition of SAP geopolymers (in g).

	P-0.8	P-1	P-1.5	P-2	P-3	P-4
Metakaolin	300	300	300	300	300	300
H_3_PO_4_	346	277	184	139	92	69
Water	50	90	100	110	130	140

**Table 4:** Degree of crystallinity of SAP geopolymers (%).

P-0.8	21
P-1	2
P-1.5	3
P-2	3
P-3	4
P-4	4

## Data Availability

Dataset available on request from the authors.

## Abbreviations

The following abbreviations are used in this manuscript:

amb.	Ambient conditions
CS	Compressive strength
EDS	Energy dispersive X-ray spectroscopy
FTIR	Fourier-transform infrared spectroscopy
HT	High-temperature
IC	Isothermal calorimetry
MAS NMR	Magin angle spinning nuclear magnetic resonance spectroscopy
MIP	Mercury intrusion porosiemetry
MK	Metakaolin
RT	Room temperature
SAP	Silico-alumino-phosphate geopolymer
SAXS	Small angle X-ray scattering
ssNMR	Solid-state nuclear magnetic resonance spectroscopy
TA	Thermal analysis
XRF	X-ray fluorescence spectroscopy
XRD	X-ray diffraction
